# Clinical Features and Laboratory Diagnosis of Infection with the Potential Bioterrorism Agents *Burkholderia Mallei* and *Burkholderia Pseudomallei*

**Published:** 2007-09

**Authors:** Jacob Gilad, David Schwartz, Yoram Amsalem

**Affiliations:** 1 *CBRN Medicine Branch, Israel Defense Forces Medical Corps, Tel-Hashomer;*; 2 *Microbiology Laboratory, Tel-Aviv Sourasky Medical Center, Tel-Aviv, Israel*

**Keywords:** melioidosis, glanders, identification, bioterrorism, preparedness, *Burkholderia mallei*, *Burkholderia pseudomallei*, human infection

## Abstract

*Burkholderia mallei* and *Burkholderia pseudomallei* are the causative organisms of Glanders and Melioidosis, respectively. Although now rare in Western countries, both organisms have recently gained much interest because of their unique potential as bioterrorism agents. These organisms are less familiar to medical and laboratory personnel than other select bioterrorism bacterial agents and thus heightened awareness of Glanders and Melioidosis is crucial in order to enable adequate emergency preparedness and response to deliberate release of *B. mallei* and *B. pseudomallei*. The microbiological diagnosis of both species in the clinical laboratory is complicated. This paper reviews the various challenges and pitfalls associated with the diagnosis of Melioidosis and Glanders in the clinical setting, with emphasis on the role of sentinel laboratories.

## INTRODUCTION

*Burkholderia mallei* and *Burkholderia pseudomallei* are the causative organisms of Glanders and Melioidosis, respectively. Although now rare in Western countries, both organisms have recently gained much interest because of their unique potential as bioterrorism agents (1). Despite being unique organisms, *B. mallei* and *B. pseudomallei* share many similarities and should be considered together in the context of a deliberate release event.

*B.mallei* and *B. pseudomallei* are less familiar to medical and laboratory personnel than other select bioterrorism bacterial agents such as *Bacillus anthracis*, *Yersinia pestis* and *Francisella tularensis* and thus heightened awareness of Glanders and Melioidosis is crucial in order to enable adequate emergency preparedness and response to deliberate release of *B. mallei* or *B. pseudomallei*. Recognition of the principles of microbiological diagnosis of Glanders and Melioidosis is of utmost importance, in settings with little or no experience with these infectious diseases. This review will focus on the clinical features and diagnostic challenges and pitfalls associated with these agents in the clinical microbiology laboratory.

## OVERVIEW OF MELIOIDOSIS AND GLANDERS

### Melioidosis

Most cases of melioidosis currently occur in endemic regions in Southeastern Asia and Oceania, especially Northern Australia, Thailand, Singapore, Vietnam, Malaysia and Burma, while cases in Western countries mainly involve reactivation or recent travel to endemic areas (2). In Australia, melioidosis is particularly common in the Northern Territory and to a lesser extent in Western Australia and Queensland (3). In Thailand, melioidosis is also endemic in the northern part of the country (4).

Melioidosis affects both humans and animals. The main reservoir for *B. pseudomallei* is the contaminated environment, especially soil and water (5, 6). Various animals may contract melioidosis and serve as a reservoir for continued epizootic infection (2). Human-to-human and animal-to-human transmission is considered extremely rare. *B. pseudomallei* has a saprophytic nature and are capable of surviving in a relatively hostile environment, but also demonstrates significant surviving capabilities during its interaction with the host immune system (7).

Humans (and animals) acquire melioidosis through percutaneous inoculation, inhalation or ingestion, and more rarely sexual transmission. The percutaneous route is thought to be the predominant portal of entry, even for patients with pneumonic melioidosis, but pulmonary infection may occur directly via inhalation (8) and has also been reported in the context of near-drowining following the Tsunami in Thailand (9). Host risk factors play a major role in the acquisition of melioidosis and nearly two-thirds of patients with naturally-occurring melioidosis had recognized risk factors, mainly diabetes mellitus, chronic renal or lung disease and alcohol abuse (10, 11). Nevertheless, the natural history of exposure to *B. pseudomallei* in a deliberate release scenario is not well understood.

The clinical manifestations of melioidosis are protean and primary infection and suppurative complications may involve virtually every body organ. A substantial percentage of melioidosis cases have bacteremia (40-60%) with septic shock present in one-fifth, and appreciable mortality (up to 60%) (10, 12, 13). Pneumonia is by far the most common syndrome associated with *B. pseudomallei* infection and in endemic areas, melioidosis may be the most common cause of community-acquired pneumonia (14). Pneumonia in melioidosis may be similar to acute pneumonia but may also manifest as subacute or chronic disease resembling tuberculosis (8). Pulmonary reactivation may occur uncommonly, up to 30 years from initial infection (15).

Apart from pulmonary infection, melioidosis may affect every organ system, resulting in genitourinary infection (including prostatic infection) (10), suppurative parotitis (16), various forms of central nervous system infection (10), osteomyelitis and septic arthritis, intra-abdominal abscess formation mainly involving the spleen, liver or adrenals, nectrotizing skin infection, mycotic aneurysms or pericarditis and corneal abscesses (8).

The outcome of melioidosis is related to host risk factors, adequate therapy and disease severity; mortality is <10% in isolated bacteremia or localized disease without bacteremia, up to 40% with septicemic disease involving at least one focus (e.g. pneumonia) with bacteremia, and 40-90% when severe sepsis or septic shock occur (17).

### Glanders

Glanders primarily affects animals and can be transmitted both from animal-to-animal and animal-to-human, while human-to-human transmission is rare, at least in nature. Most human cases during the 20^th^ century were occupational infections among laboratory workers, horse handlers, butchers and veterinarians (8). Glanders has also been implicated in the first modern attempt of biological warfare when used by the Germans against horses in The Great War (18). With quarantine and veterinary control, by 1939, glanders has been eliminated from most parts of Western Europe and North America (19). Enzootic foci continue in South America, the Middle East, Africa and Asia.

An occupational *B. mallei* infection had occurred recently (year 2000) in the United States and involved a microbiology laboratory worker (20). The patient developed fever and lymphadenopathy, followed by diabetic ketoacidosis and intra-abdominal abscess formation and was successfully treated with antibiotics. Interesting points reflected from this case are: (a) Acquisition of *B. mallei* infection due to inadequate safety precautions while working with the organism; (b) Difficulty in diagnosing clinical *B. mallei* infection; and (c) Misidentification of the clinical isolate by routine laboratory methods.

*B. mallei* shares many genetic elements with *B. pseudomallei* and both organisms have a similar allelic profile (21). It is not an environmental pathogen and its main reservoir is animals and it primarily causes a disease of equids. The mode of infection in glanders is not at all clear and probably includes inhalation, percutaneous inoculation and ingestion. Purulent discharge from infected animal respiratory tract or skin is highly contagious (8) and is a prime route of natural transmission among horses. The incubation period may range from 1-5 days (in inhalational infection) to many months. Reactivation has been described as in meliodosis.

There are two types of clinical infection in equids (19). Glanders occurs through inhalation or more commonly ingestion whereas Farcy probably occurs through direct inoculation. In glanders, there is an acute or chronic lung infection spanning the whole upper and lower respiratory tract, and dissemination with multiple abscess formation. Farcy on the other hand, appears as swelling in skin and subcutaneous tissues that ulcerate. The surrounding lymphatic vessels become hard and enlarged as are regional lymph nodes (farcy pipes and farcy buds) (22).

Human glanders, if acquired by the inhalational route, can produce fever, ulcerative necrosis of the upper and lower respiratory tract with purulent nasal discharge, extensive pneumonia, cervical or mediastinal lymphadenopathy and pustular skin lesions (which may resemble smallpox). Prostration, out of proportion to clinical signs is a classic finding (19). Septicemia follows with involvement of various internal organs, such as in melioidosis. Without treatment, death almost invariably occurs within 10 days. Chronic human glanders is associated with multiple subcutaneous and intramuscular abscesses, lymphadenopathy and lymphangitis and involves half of naturally-occurring infections, and unless a very low inoculum is involved, melioidosis and glanders in the context of deliberate release may thus be indistinguishable from one another (22).

## CLINICAL LABORATORY DIAGNOSIS

### Initial work-up

The genus *Burkholderia* contains more than 20 valid species, most of which are environmental bacteria isolated from soil or water. Three main human pathogens recognized in this group are *B. mallei*, *B. pseudomallei* and *B. cepacia*. Uncommonly, infection may occur with rare taxons such as *B. fungorum*, *B. gladioli* (previously *B. cocovenenans*) and *B. thailandensis*. *Burkholderia* species are aerobic, non-spore-forming, gram-negative bacilli. All species except *B. mallei* are motile due to the presence of polar flagella. All grow on MacConkey agar and appear as non-fermenters (23). Owing to their ability to survive in hostile environments, standard specimen collection and transport principles are sufficient for recovering *Burkholderia* species in clinical practice. Methods for isolating and identifying *Burkholderia* may include culture-based, antibody/antigen-based and molecular-based techniques.

On gram-stain all species may appear similar; however, *B. pseudomallei* may show a typical bipolar staining and a safety-pin appearance (23) while *B. mallei* may appear as coccobacilli (24). All organisms grow well on MacConkey, blood and chocolate agars, although *B. mallei* are somewhat more fastidious and may not grow easily on MacConkey. Moreover, growth in standard blood culture bottles occurs within less than 5 days and therefore extended incubation is not required. With the BacT/Alert system, 90% of *B. pseudomallei* strains grew within 48 hours (25). It has been shown that bacterial count in blood correlates with outcome in melioidosis (26). *B. mallei* on the other hand is rarely isolated from blood during the initial presentation (22).

Improved isolation of *B. pseudomallei* can be achieved by using the Ashdown agar medium which contains tryptic soy agar with glycerol, crystal violet, neutral red and gentamicin. Ashdown medium may be especially useful for specimens from non-sterile sites such as throat or rectum as well as sputum (27). Newer modified agar media (e.g. with colistin) are now available (28). An enrichment broth containing Ashdown medium can also be used for bedside sample inoculation (29). A recent study evaluating the utility of throat cultures in melioidosis has found a sensitivity of 36% (24% for sputum-negative and 79% for sputum-positive patients) (30) for diagnosing melioidosis. Recovery rate was higher with selective broth than selective agar.

Newer proposed media for *B. pseudomallei* isolation include the *B. pseudomallei* selective agar and *B. cepacia* medium. The selective agar is thought to promote growth of mucoid *B. pseudomallei* colonies as compared to the Ashdown medium (31). Neither is routinely used in laboratories outside endemic areas. A comparative study of the three selective media, showed an equal sensitivity for all media, except that at 24 hours, colonies on Ashdown agar were much smaller. Colony counts were maximal with *B. cepacia* medium. *B. pseudomallei* selective agar was the least selective of all media. Of note is that *B. cepacia* medium is also suitable for isolating *B. mallei* and therefore may be most appropriate for diagnosis in a deliberate release incident.

Growth of *B. pseudomallei* in the first two days reveals smooth colonies, sometimes yellow to orange-pigmented with a putrid odor, that after several days form dry wrinkled colonies that resemble *Pseudomonas stutzeri*. Growing organisms produce a distinctive earthy and musty odor. On Ashdown medium colonies are colored deep pink. If *B. pseudomallei* is suspected, key phenotypic features include the ability to grow in 42°C, motility, oxidase activity and nitrate reduction. On the other hand, *B. mallei* is non-motile, non-flagellated, does not grow in 42°C and has variable oxidase activity. *B. mallei* colonies are smooth, grey and translucent, without any distinctive pigment or odor. *B. pseudomallei* can be differentiated from P. stutzeri by number of flagella (the latter has only one) and arginine dihydrolase activity that is positive only with *B. pseudomallei* (32).

Isolates suspected as *B. mallei* or *B. pseudomallei* should immediately be referred to a specialized laboratory and not further investigated at the level of the sentinel laboratory. However, if not initially suspected, the possibility for *B. mallei* or *B. pseudomallei* should be raised if several practical key criteria are met during the routine identification process of gram-negative clinical isolates (Table 1). At such instance, isolates should be immediately referred after proper arrangements are made to a reference laboratory.

**Table 1:** Key phenotypic features that should raise the suspicion for *B. mallei* or *B. pseudomallei* in the laboratory

Key feature	*B. mallei*	*B. pseudomallei*

Gram-stain morphology	Gram-negative coccobacilli	Bipolar gram-negative bacilli
Growth on medium^a^	Growth on blood agar within 24-48 hours; delayed or no growth on MacConkey agar	Growth on blood / MacConkey agar within 24-48 hours;
Morphology of colonies	Grey; translucent; smooth; no pigment	White; smooth; yellow / no pigment; aging colonies become wrinkled;
Odor	Odorless	Earthy and musty
Motility	Non-motile	Motile
Cytochrome oxidase	Variable	Positive
Indole Production	Negative	Negative
Polymyxin B	Resistant	Resistant
Nitrate reduction to gas	Negative	Positive
Sugar utilization	Non-fermenter	Non-fermenter

a Both species grow well in 37°C but only *B. pseudomallei* grows in 42°C.

### Organism identification

Identification of *B. mallei* and *B. pseudomallei* using commercial systems has been reported, including the API 20NE (bioMerieux, Marcy l’Etoile, France), Microbact 24E (Oxoid, Hampshire, United Kingdom), VITEK-1 or VITEK-2 (bioMerieux) and MicroScan WalkAway (Dade Behring, Sacramento, CA). Good results for *B. pseudomallei* identification were reported in two studies with the API20NE system (33, 34) but misidentification of almost 10% of *B. pseudomallei* isolates was reported in another (*B. pseudomallei* was mostly misidentified as *Chromobacterium violaceum*) (35). The API20NE system, however, is not designed to identify *B. mallei*. A more recent evaluation of API20NE as well as RapID NF Plus (Remel Inc, Lenexa, KS) has been carried out with different sets of human, animal and environmental *B. pseudomallei* and *B. mallei* isolates (27). *B. pseudomallei* was correctly identified in only 60% by API20NE and in the remaining cases was mainly misidentified as other non-fermenters. With the Remel system, none of the *B. pseudomallei* isolates were correctly identified. Both systems failed to identify *B. mallei* in 100% of cases. These results, along with the risks of laboratory processing of these agents in open systems such as API20NE, suggest that such systems cannot be currently recommended for *B. mallei* or *B. pseudomallei* identification in sentinel laboratories.

The earlier version of VITEK-2 gave disappointing results with correct identification of only 19% of tested isolates as *B. pseudomallei* (34). The remaining isolates were misidentified as other non-fermenters, but VITEK-1 had good results of 99% identification. A similar problem has been encountered with another automated system (Phoenix NMIC-ID4, Becton Dickinson, Sparks, MD) (37). The introduction of colorimetric identification cards to VITEK-2 instead of the fluorometric cards used originally, has improved the identification performance for non-fermenting gram-negative bacilli. Indeed, a recent evaluation of the colorimetric method has shown improved identification to a level of up to 75-80% (38). Interestingly, identification rates were dependent on the culture media used and were worse for MacConkey as compared to various blood agars. A newer version of the VITEK-2 identification system’s software is expected to increase identification rates to 97-99% (38). Of note is that in the recent case of occupational glanders described among a laboratory worker in the United States (20), an automated system misidentified *B. mallei* as *Pseudomonas fluorescens* or *Pseudomonas putida.*

### Serological diagnosis

For *B. pseudomallei*, various techniques have been used to detect specific antibodies. Antibody detection is important mainly for epidemiologic purposes and serological surveys since during clinical illness *B. pseudomallei* recovery rate are very high. The most used method is indirect hemagglutination or ELISA but this technique has many limitations, especially in endemic areas. Usually a cutoff of 1:160 or 1:80 is set in most endemic areas and 1:40 in less endemic ones (39). Conversely, during acute infection, antibodies are usually not detected, until seroconvertion occurs at a later stage.

It has been claimed that seropositivity to *B. pseudomallei* may be a result of environmental exposure to *B. thailandensis*, but a recent study did not found universal cross-reactivity between these two species. Nevertheless, marked cross-reactivity has been found between *B. mallei* and *B. pseudomallei* and therefore, serological testing cannot be used to differentiate between Melioidosis and Glanders (40). Serological testing for *B. mallei* is even more problematic. Seroconversion occurs rather late and unexplained high background titers in normal sera make interpretation difficult. A complement-fixation assay has been in use, and is considered more specific but less sensitive. Moreover, this method may not detect antibodies as late as 40 days from disease onset (22).

### Antigen testing

An alternative (although less accurate) to molecular detection of *Burkholderiaceae* is direct antigen testing using immunofluorescence or agglutination. Rapid immunofluorescent tests have been devised for detecting *B. mallei* or *B. pseudomallei* in clinical samples prior to culture. One-step immunofluorescence has been shown to be highly specific for detecting *B. pseudomallei* in clinical samples (>99%). However, sensitivity has been disappointing (66%) (41). Co-agglutination and latex agglutination with polyclonal or monoclonal antibodies against *B. pseudomallei* have also been utilized. Co-agglutination showed a 100% sensitivity and specificity when tested with blood culture supernatants whereas the respective figures for latex agglutination were 100% and 90% respectively (42). Previous studies of latex agglutination applied on cultured isolates have yielded similar results (43, 44). In addition, latex agglutination tests using monoclonal antibodies against lipopolysaccharide and exopolysaccharide have been shown to accurately differentiate *B. pseudomallei* and *B. thailandensis* in environmental specimens (45).

### Molecular methods

In recent years, molecular methods for identification of *B. mallei* and *B. pseudomallei* in clinical samples have gained much interest, due to the difficulties in identifying these organisms, especially in non-endemic areas and the biosafety level requirements for processing *B. mallei* and *B. pseudomallei* samples in classical methods (34, 35, 46). Molecular tools may be applied directly on clinical specimens for diagnosing (or ruling out) the presence of *B. mallei* or *B. pseudomallei*, or alternatively, for final identification of isolates purified from clinical samples.

Many studied have used such molecular tools but a few have clinical applications. The 16S rRNA of both *B. mallei* and *B. pseudomallei* clinical isolates were recently cloned and sequenced so that by using appropriate primers, *B. mallei* or *B. pseudomallei* may now be detected using real-time PCR in less than 12 hours (47). Other molecular protocols for differentiating *B. mallei* and *B. pseudomallei* have been recently established, relying on 23S rDNA (46), real-time PCR of type III secretion system genes (48) and TaqMan allelic-discrimination assay (49). A multiplex real-time PCR with molecular beacons for diagnosing anthrax, tularemia, plague and glanders has been recently described (50).

Most studies however, have not used *B. mallei* -specific primers but relied on the phenomenon of loss of *B. pseudomallei* -specific sequences in *B.mallei*. Recently, a *fliP*-based PCR assay has been used successfully for specifically detecting *B. mallei* infection in clinical samples from diseased horses and appears to be a promising method for diagnosing human infection as well (51). Interestingly, 16S rRNA PCR which is widely used for molecular diagnosis of bacterial infections, is of less value in the case of *B. pseudomallei* due to >99% sequence homology between *B. pseudomallei* and *B. thailandensis* and thus additional genes such as *groEL* should be subject to PCR (52). Primers that target specific nucleotide sequences in *B. mallei* have been recently shown to accurately differentiate *B. mallei* from *B. pseudomallei* and related *Burkholderiaceae* (53).

Inglis *et al*. have recently compared several methods for identification of *B. pseudomallei*, including API20NE, latex agglutination, cell-wall fatty acid analysis and PCR (54). PCR had 100% sensitivity and specificity while the API20NE had an unacceptable sensitivity (37%) and specificity was only 92%. The authors recommended using a rapid agglutination test in the initial work-up (94% sensitivity and 83% specificity) and confirmation using PCR. A noted drawback of the agglutination assay is difficulty in reading the test in a biosafety cabinet.

## ANTIMICROBIAL SUSCEPTIBILITY TESTING

Antimicrobial susceptibility of *Burkholderiaceae* has been evaluated in the past according to Clinical and Laboratory Standards Institute (CLSI) breakpoints for *Pseudomonas* species. Recently, CLSI recommendations for potential bioterrorism agents have included *B. mallei* and *B. pseudomallei*. In general, disk-diffusion is not recommended for *Burkholderiaceae* (55) and the minimal inhibitory concentration (MIC) should be determined. E-test is generally considered a reliable alternative to broth dilution (23), but at least for *B. mallei*, differences in MIC between E-test and dilution methods have been observed (56). Like with other non-fermentative gram-negative bacilli, *in vitro* susceptibility and *in vivo* outcome do not always correlate.

Therapy of Melioidosis requires an intensive-phase of bactericidal agents (mainly ceftazidime-based or carbapenem-based regimens) and maintenance therapy with a combination of oral agents, usually including trimethoprim-sulfamethoxazole, doxycycline and chloramphenicol (57). No firm data exists regarding treatment of Glanders but current consensus recommends a similar protocol to that of Melioidosis (22). Per CLSI (55), susceptibility should be determined after inoculation to cation-adjusted Mueller-Hinton agar or broth medium. Quality Control is achieved with a control strain of *Pseudomonas aeruginosa*. Available MIC breakpoints (expressed in μg/ml) for the susceptible, intermediate-resistance and resistant categories, are presented in Table 2.

**Table 2:** Clinical and Laboratory Standards Institute (CLSI) Antimicrobial susceptibility breakpoints for *Burkholderia mallei* and *Burkholderia pseudomallei*

Agent	Organism	MIC (μg/Ml)		
		*Susceptible*	*Intermediate*	*Resistant*

Amoxicillin-clavulanate	*B. pseudomallei*	≤8/4	16/8	≥32/16
Ceftazidime	*B. pseudomallei*	≤8	16	≥32
	*B. mallei*	≤8	16	≥32
Tetracycline / Doxycycline	*B. pseudomallei*	≤4	8	≥16
	*B. mallei*	≤4	8	≥16
Trimethoprim-sulfamethoxazole	*B. pseudomallei*	≤2/38	-	≥4/76
Imipenem	*B. pseudomallei*	≤4	8	≥16
	*B. mallei*	≤4	8	≥16

Primary resistance to ceftazidime and amoxicillin-clavulanate has not been observed with *B. pseudomallei*, and emergence of resistance during therapy is rare (58). Seven genes encoding for extended-spectrum beta-lactamases have been detected to date (59), the most important of which is BPS-1 which inactivates cephalosporins but is inhibited by clavulanate (60, 61). Ceftazidime resistance may be acquired through a class D enzyme, such as OXA-42 or OXA-43 (62) or reduced sensitivity to beta-lactamase inhibitors (63). Resistance to other antimicrobials used for treating *B. pseudomallei*, such as doxycycline, chloramphenicol, trimethoprim-sulfamethoxazole is thought to be uncommon (<10%) (64) and natural resistance to carbapenems also remains uncommon in *B. pseudomallei* (65). Fluoroquinolones and aminoglycosides show poor activity against *B. pseudomallei* (66). A recent study has also shown universal susceptibility to the new broad-spectrum agent tigecycline (67). A post-antibiotic effect which is especially important in eradication of intra-cellular organisms has been shown to exist only with carbapenems and fluoroquinolones but not ceftazidime and amoxicillin-clavulanate (68).

The antibiotic susceptibility pattern of *B. mallei* is generally similar to *B. pseudomallei*. A recent survey of 15 *B. mallei* isolates has shown 100% susceptibility of *B. mallei* to cefotaxime, ceftazidime, amoxicillin-clavulanate, piperacillin-tazobactam, imipenem, chloramphenicol, trimethoprim-sulfamethoxazole and tetracyclines (66). MIC values in general were lower than those of *B. pseudomallei*, within the susceptible breakpoint range. Another study of 11 strains also found notable susceptibility of *B. mallei* to fluoroquinolones (69). Frequent susceptibility to aminoglycosides has also been reported (69, 70) but the intra-cellular nature of *B. mallei* may render aminoglycoside therapy ineffective.

## CONCLUSIONS

*B. mallei* and *B. pseudomallei* are now included in formal emergency preparedness plans and guidelines issued by various authorities in the United States and Europe (1, 22, 24, 71). The actual risk for deliberate release *B. mallei* or *B. pseudomallei* is unknown, at least publicly, and therefore most efforts are concentrated on efficiency and safety of laboratory-based diagnosis of Melioidosis and Glanders.

Currently, the diagnosis of *B. mallei* and *B. pseudomallei* in the clinical laboratory is highly problematic, given the low awareness of physicians to the clinical manifestations of Melioidosis and Glanders, lack of experience among microbiologists outside endemic areas, lack of appropriate media and identification systems in the average sentinel laboratory and the biosafety conditions necessary to process these organisms (Level 2 for processing of clinical samples and Level 3 for processing of clinical isolates) (1).

Until new or improved diagnostic modalities become available at the level of the sentinel laboratory, isolates compatible with *B. mallei* or *B. pseudomallei* should be referred immediately to a reference laboratory. Sentinel laboratory work-up should consist of the minimal phenotypic and biochemical tests that suggest this diagnosis. In cases where a high clinical index of suspicion exists, direct referral of clinical samples to a specialized setting may be most appropriate.
